# Factors associated with tuberculosis treatment adherence: reflections on a retrospective cohort study in Lesotho

**DOI:** 10.11604/pamj.2026.54.10.53079

**Published:** 2026-05-14

**Authors:** Gianella Ramos Mejia

**Affiliations:** 1 Instituto de Investigaciones en Ciencias Biomédicas, Universidad Ricardo Palma, Lima, Perú

**Keywords:** Tuberculosis, treatment adherence, public health

## Reaction

I read with great interest the article on tuberculosis treatment adherence and associated factors in the district of Butha-Buthe, Lesotho [1], which provides relevant evidence on the determinants of therapeutic adherence in a high-burden setting. I believe this study makes a significant contribution to understanding the sociodemographic and clinical factors involved in adherence to anti-tuberculosis treatment.

The authors identify several factors associated with adherence, highlighting the influence of sociodemographic variables and health system-related aspects. This finding is consistent with global reports, where the World Health Organization has emphasized that adherence to tuberculosis treatment remains a critical challenge, particularly in developing countries [2]. In this regard, factors such as educational level, social support, access to healthcare services, and economic conditions have been widely described as key determinants of treatment adherence [3,4].

However, I believe it is important to further examine certain methodological aspects of the study. Given its retrospective cohort design, there is a potential for information bias and limitations related to the quality of recorded data, which could influence the estimation of associated factors. Additionally, the absence of variables related to mental health, social stigma, or family support may limit a comprehensive understanding of adherence, as these factors have shown a significant impact in different settings [5,6].

Furthermore, the findings of this study gain particular relevance when compared with the situation in other regions. In Latin America, and especially in Peru, tuberculosis remains a major public health concern, where treatment adherence continues to be a significant challenge despite the implementation of strategies such as directly observed therapy (DOTS) [7]. Studies conducted in similar contexts have shown that treatment default is frequently associated with socioeconomic factors, substance use, and barriers to accessing healthcare services [8].

**Figure 1:**
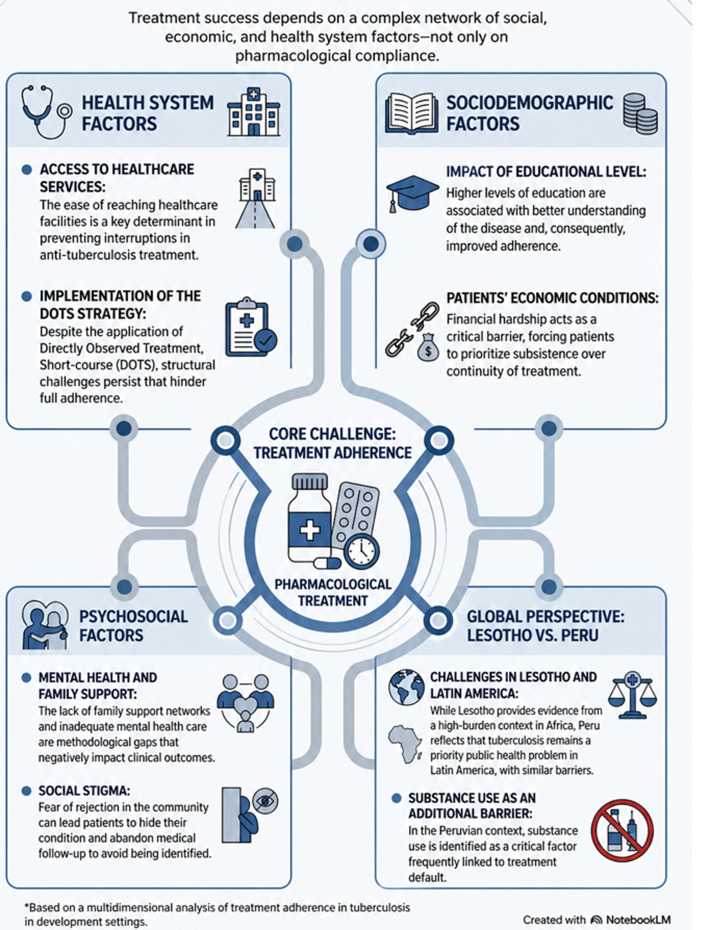
multidimensional determinants of tuberculosis treatment adherence, including health system, sociodemographic, and psychosocial factors, and their interaction with pharmacological treatment

Based on the above, I consider that the study conducted in Lesotho represents a valuable contribution that reinforces the need to address tuberculosis treatment adherence from a multidimensional perspective. It is essential to integrate interventions that not only focus on pharmacological treatment but also consider social, educational, and psychological determinants (Figure 1). Likewise, future research should incorporate additional variables and prospective designs to allow for a better assessment of causal factors.

Finally, I congratulate the authors on their contribution and believe that their findings may serve as a basis for developing more effective strategies aimed at improving treatment adherence and, consequently, tuberculosis control at a global level.
